# Molecular detection and genetic variability of *Ehrlichia canis* in pet dogs in Xinjiang, China

**DOI:** 10.14202/vetworld.2020.916-922

**Published:** 2020-05-18

**Authors:** Qiao Mengfan, Wang Lixia, Lei Ying, Ren Yan, Cai Kuojun, Zhang Jinsheng, Zhang Zaichao, Yu Weiwei, Peng Yelong, Cai Xuepeng, Li Chongyang, Qiao Jun, Meng Qingling

**Affiliations:** 1 Department of Agriculture, College of Animal Science and Technology, Shihezi University, Shihezi, Xinjiang, 832003, China; 2 Department of Life Sciences, School of Biotechnology, Central South University, Changsha, Hunan, 410012, China; 3 Department of Medicine, School of Medicine, Southeast University, Nanjing, Jiangsu, 211189, China; 4 Department of Medicine, School of Medicine, Shihezi University, Shihezi, Xinjiang, 832003, China; 5 Center for Animal Disease Prevention and Control, Urumqi, Xinjiang, 830000, China; 6 Center for Animal Disease Prevention and Control, Tacheng, Xinjiang, 834700, China; 7 Center for Animal Disease Prevention and Control, Changji, Xinjiang, 831500, China; 8 Bazhou Center for Animal Disease Prevention and Control, Korla, Xinjiang, 841000, China; 9 Center for Animal Disease Prevention and Control, Aksu, Xinjiang, 843000, China; 10 State Key Lab of Veterinary Etiological Biology, Lanzhou Veterinary Research Institute, Chinese Academy of Agricultural Sciences, Lanzhou, Gansu, 730046, China

**Keywords:** *Ehrlichia canis*, genetic characteristics, *gp36*, pet dog, *Rhipicephalus sanguineus sensu lato*

## Abstract

**Background and Aim:**

As a tick-borne zoonotic pathogen, *Ehrlichia canis* has already posed a threat to public health and safety. This study aimed to clarify the prevalence and molecular characteristics of *E. canis* in pet dogs in Xinjiang, China.

**Materials and Methods:**

A total of 297 blood samples of pet dogs and 709 skin ticks (*Rhipicephalus sanguineus sensu lato*) were subjected to molecular detection using PCR for *E. canis* 16S *rRNA* gene, and then, positive samples were amplified, sequenced, and phylogenetically analyzed for *E. canis* *gp36* gene.

**Results:**

The PCR detection showed that the positive rate of PCR was 12.12% (36/297) in blood samples and 15.23% (108/709) in tick samples, respectively. Based on the phylogenetic analysis of *E. canis* *gp36* protein, these *E. canis* strains in different geographical regions of the world can be divided into Genogroup I and Genogroup II. Among them, the Xinjiang epidemic strain XJ-6 and 533, 36, 1055, Kasur1, and Jake strains were clustered into subgroup 1.1 of Genogroup I, while the XJ-2, XJ-21, and XJ-35 strains and the TWN1, TWN4, CM180, and CM196 strains were closely related and belonged to subgroup 2.2 of Genogroup II, displaying high genetic diversity.

**Conclusion:**

This is the first study focusing on the molecular epidemiology of *E. canis* infection in pet dogs, which revealed that *E. canis* infection had been occurred in Xinjiang, China. More importantly, this study confirmed that the substantial variability in immunoreactive protein *gp36* from *E. canis* strains circulating in pet dogs.

## Introduction

*Ehrlichia canis* is the pathogen causing canine monocytic ehrlichiosis (CME). As a member of the strict intracellular parasitic Gram-negative microorganism in the family Anaplasmataceae [1], *E. canis* is mainly transmitted by ticks (*Rhipicephalus sanguineus sensu lato*). Dog infected with *E. canis* is characterized by fever, depression, anorexia, increased ocular and nasal secretions, thrombocytopenia, and anemia, which are harmful to dogs [2,3]. Since *E. canis* was first detected in Algeria in 1935, it has been found in dogs and ticks in North America, Europe, Asia, and Africa. At present, CME is spreading all over the world [3-7].

Dogs are important companion animal of human beings, which are part of our daily lives. With the increasing number of pet dogs in cities, however, the more frequent exposures and interactions between companion animals and humans increase the infection risks of zoonotic diseases of human beings. In recent years, some important zoonoses (e.g., rabies and rickettsiosis) associated with pet dogs have received more and more attention from medical and veterinary researchers [8,9]. Therefore, as a newly discovered zoonotic pathogen, *E. canis* infection poses a threat to public health and safety [8]. Meanwhile, *E. canis* infection is closely related to the distribution and epidemic of *Rhipicephalus sanguineus sensu lato*. It was also found that the immunogenic protein *gp36* of *E. canis* strains from different geographical regions of the world existed great genetic variation and showed obvious genetic diversity [10-15].

Xinjiang, located in Northwest China, is an area where *E. canis* vectors, *Rhipicephalus sanguineus sensu lato*, are populated, and is also one of the important focuses of tick-borne diseases. However, the infection status and molecular characteristics of *E. canis* in pet dogs are still unclear.

The main purpose of this study was to investigate the infection status and explore the molecular characteristics of *E. canis* in pet dogs in Xinjiang, China. Here, the blood samples were collected from pet hospitals in Xinjiang and the ticks parasitized on dog surface were detected by PCR, and the genetic evolution of *E. canis* Xinjiang epidemic strains was analyzed to reveal the genetic evolution relationship between *E. canis* epidemic strains from Xinjiang, China and other regions over the world, which will provide valuable epidemiological data for the prevention and control of *E. canis* infection.

## Materials and Methods

### Ethical approval

The experiments were carried out in accordance with the guidelines issued by the Ethical Committee of Shihezi University.

### Source and collection of samples

**Supplementary Figure-1:**
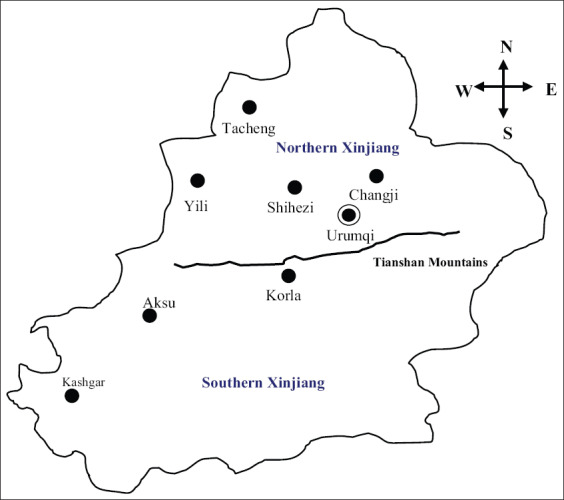
Geographic locations of the investigated regions in Xinjiang, [Source: www.zhongguolu.com/xinjiang/].

During March 2016-March 2019, a total of 297 blood samples of dog and 709 samples of ticks (*Rhipicephalus sanguineus sensu lato*) on the skin surface of pet dogs were collected from pet hospitals in eight different geographical regions of Xinjiang (Tacheng, Yili, Shihezi, Urumqi, Changji, Korla, Aksu, and Kashgar) (Supplementary Figure-1). The collected clinical samples were sealed and placed in an icebox to transport at 4°C to the key laboratory of animal disease prevention and control in Xinjiang.

### Primer design

Briefly, 16S *rRNA* and *gp36* gene sequences of *E. canis* deposited in GenBank (http://www.ncbi.nlm.nih.gov/genbank/) were downloaded and compared, and the conservative sequences were selected. The specific primers for 16S *rRNA* and *gp36* gene were designed by Premier 5.0 primer software according to the conservative region, respectively. 16S *rRNA* FP1-RP1 primer was used for PCR detection of whole blood and tick samples, while *gp36* FP2-RP2 primer was used for the amplification of *gp36* gene of *E. canis* epidemic strains in Xinjiang (Table-1). The primers were synthesized by BGI Group (BGI, China).

**Table-1:** List of primer sequences used in this study.

Primer name	Nucleotide sequence (5’®3’)	Target gene	Size of product (bp)
16S-FP1	CCTACGTTAGATTAGCTAGTTG	*16S rRNA*	465
16S-RP1	CTGGTGTTCCTCCTAATATCTA		
*gp36*-FP2	atgctatttatactaatgggttat	*gp36*	845-865
*gp36*-RP2	TTAGTACAACCAGTTAGGCATATCAG		

### DNA extraction from samples

In brief, DNA was extracted from blood samples of pet dogs using cell genomic DNA extraction kit (TaKaRa, Japan). Tick samples from pet dogs were identified by inverted microscopy and then placed in a grinder (one sample per tick) and grinded with 0.5 mL sterilized saline. The milling fluid was collected in 1.5 mL EP tube and frozen and thawed 3 times. Then, tick’s DNA was extracted according to the instructions of DNA extraction kit (Qiagen, Germany).

### Molecular detection of *E. canis* by PCR

Briefly, PCR detection of *E. canis* was performed using 16S *rRNA* FP1-RP1 primers. The PCR reaction was carried out in 50 μl reaction mixture containing H_2_O_2_ 21 uL, 16S *rRNA* FP1-RP1 primers 2 uL (0.2 μmol/L), 2×Premix Ex Taq (TaKaRa, Japan) 25 uL, and DNA template 2 uL. The PCR thermocycling conditions were as follows: Pre-denaturation at 95°C for 5 min, denaturation at 95°C for 20 s, annealing at 60°C for 30 s, elongation at 72°C for 30 s, 35 cycles, and followed by final elongation at 72°C for 10 min. Then, PCR product was detected by electrophoresis on 2% agarose gel containing ethidium bromide (TaKaRa, Japan) and was visualized using the Gel Doc XR System 2000 (BioRad, USA). The detection results of PCR were further analyzed statistically.

### Amplification of *gp36* gene in Xinjiang epidemic strain of *E. canis*

The samples with positive PCR results were selected, and the next PCR amplification of complete *gp36* gene of *E. canis* was performed using *gp36* FP2-RP2 primers. The PCR reaction system is the same as described in 1.4. PCR thermocycling conditions were as follows: Pre-denaturation at 95°C for 5 min, denaturation at 95°C for 20 s, annealing at 60°C for 30 s, extension at 72°C for 50 s; 40 cycles, and final extension at 72°C for 10 min. Then, PCR product was detected by 1.5% agarose gel electrophoresis.

### Cloning and sequencing of *gp36* gene of *E. canis* Xinjiang strain

In brief, the PCR product was extracted by DNA Gel Extraction Kit (TaKaRa, Japan) and cloned into pMD18-T vector (TaKaRa, Japan) for sequencing. Three positive clones were selected for each sample and each clone was sequenced (Sangon, China) 3 times. These sequences with completely identical sequencing in positive clone were submitted to GenBank and used for sequence comparison analysis.

### Phylogenetic analysis of different geographical strains of *E. canis* based on amino acid sequences of *gp36* protein

DNAStar 7.1 (DNASTAR Inc., USA) and Clustal X 2.1 (http://www.clustal.org/) software were used to compare the nucleotides and deduced amino acid sequences of the Xinjiang strains of *E. canis* with those of different geographical strains. The identities and genetic variations of the amino acid sequences of *gp36* protein were analyzed. Using MEGA 6.0 software (https://www.megasoftware.net/), a phylogenetic tree (Bootstrap value of 1000) was constructed by neighbor-joining method to explore the genetic relationship among different geographical strains.

### Statistical analysis

Statistical analysis was performed by SAS software (Version 9.1, SAS Institute, Inc., Cary, NC). The PCR-positive rates in different geographical regions in Xinjiang were compared using Chi-square test. p<0.05 was considered statistically significant, while p<0.01 was considered extremely significant difference.

## Results

**Figure-1:**
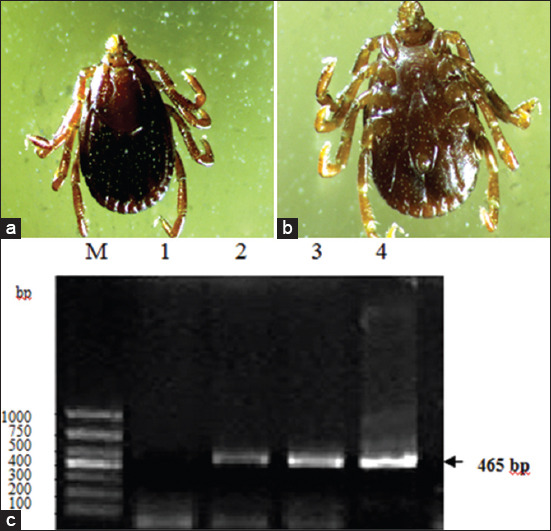
Morphological identification of *Rhipicephalus sanguineus* *sensu lato* (a) from pet dogs and molecular detection of *Ehrlichia canis 16S rRNA* gene (b). (a) Back (♀); (b) ventral (♀); (c) amplification of *E. canis 16S rRNA* gene. M, DNA marker DL 1000 (1000, 750, 500, 400, 300, 200, and 100); 1, negative samples; 2-4, positive samples.

In 297 pet blood samples and 709 *Rhipicephalus sanguineus* *sensu lato* from dogs, the total positive rate of PCR was 12.12% (36/297) and 15.23% (108/709), respectively (Table-2 and Figure-1). The results showed that the positive rate in ticks was higher than that of blood samples, and there were significant differences in the positive rate in different geographical areas, especially in Yili and Shihezi regions (p<0.05).

**Table-2:** Molecular detection of *E. canis* in different geographical regions in Xinjiang, China.

Region/location	Blood of pet dogs			Ticks from pet dogs		
	
	No. of blood samples	No. of positive	Positive rate (%) of *E. canis*	No. of tick samples	No. of positive	Positive rate (%) of *E. canis*
Tacheng	28	2	7.14 (2/28)^a^	89	9	10.11 (9/89)^a^
Yili	21	5	23.81 (5/21)^b^	106	29	27.36 (29/106)^b^
Shihezi	56	11	19.64 (11/56)^b^	102	18	17.65 (18/102)^b^
Urumqi	66	6	9.09 (6/66)^a^	123	12	9.76 (12/123)^a^
Changji	32	5	15.63 (5/32)^a^	82	9	10.98 (9/82)^a^
Korla	28	3	10.71 (3/28)^a^	71	11	15.49 (11/71)^a^
Aksu	39	2	5.13 (2/39)^a^	94	13	13.83 (13/94)^a^
Kashgar	27	2	7.41 (3/27)^a^	42	7	16.67 (7/42)^a^
Total	297	36	12.12 (36/297)	709	108	15.23 (108/709)

The different letter in same column means significant difference (p<0.05). *E. canis=Ehrlichia canis*

**Supplementary Figure-2:**
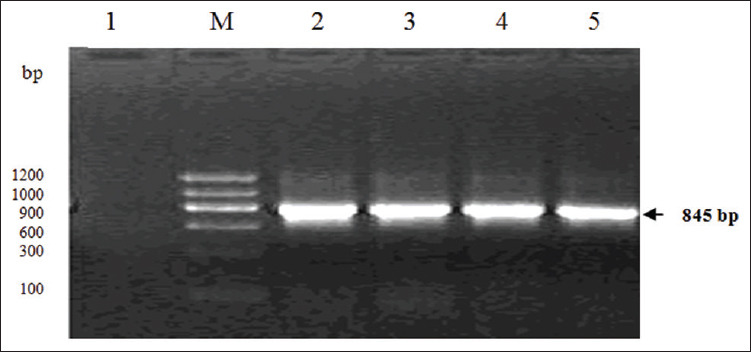
Amplification of *gp36* gene of *Ehrlichia canis* from positive blood samples of pet dogs. (M) DNA marker DL 1200 (1200, 1000, 900, 600, 300, and 100); (1) Negative samples; (2) *E. canis* XJ-2 strain; (3) *E. canis* XJ-21 strain; (4) *E. canis* XJ-35 strain; (5) *E. canis* XJ-6 strain.

**Figure-2:**
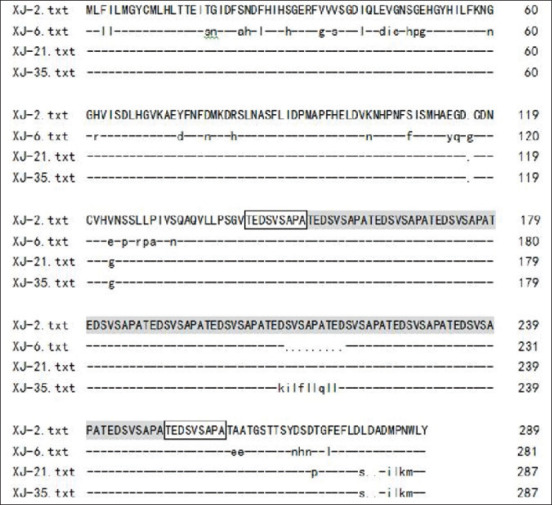
Alignments of amino acid sequences of *gp36* protein among Xinjiang strains of *Ehrlichia canis*. The tandem repeat region in amino acid sequences of *gp36* protein of *E. canis* was boxed and shadowed.

Four *gp36* genes of Xinjiang epidemic strains of *E. canis* were successfully amplified from 36 positive samples of pet dogs and sequenced (Supplementary Figure-2). The *gp36* gene sequences of four strains had been submitted to GenBank under the accession numbers (XJ-2, MN366176; XJ-6, MN366177; XJ-21, MN366178; and XJ-35, MN366179), respectively. By comparing the amino acid sequences of *gp36* protein of 42 strains in different geographical regions (Supplementary Table-1 can be obtain from the author), it was found that the genetic variation of *gp36* protein was obvious, which shared 65.71-99.26% identities in amino acid sequence (Supplementary Table-2 can be obtain from the author). Among the four Xinjiang epidemic strains, they shared 93.21% and 90.58% identities in nucleotide and amino acid sequence, respectively. However, the strain XJ-2 and XJ-21 contained 13 conserved amino acid tandem repeat units (TRR) (TEDSVSAPA), while the XJ-6 strain lacked one TRR sequence, which only contained 12 TRRs. Notably, XJ-35 strain contained 12 TRRs and a KILFLLQLL mutation sequence (Figure-2). By contrast, the Thailand strain CM180 and CM196 contained 8 and 13 TRRs in amino acid sequences of *gp36* protein, respectively, while the Israel strain (strain 611) uniquely possessed TRR of TEDPVSATA.

**Figure-3:**
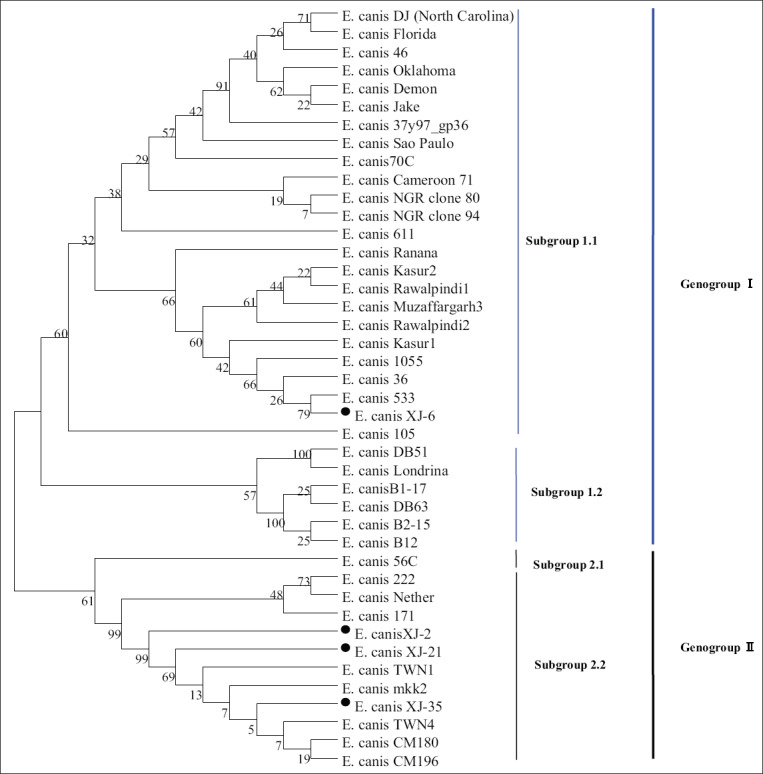
Phylogenetic analysis of different geographical strains of *Ehrlichia canis* based on amino acid sequences of *gp36* protein. The amino acid sequences of *gp36* protein of *E. canis* obtained in this study and available in GenBank were used to construct phylogenetic tree by the neighbor-joining method. Bootstrap values were calculated with 1000 replicates. Filled circle indicates *E. canis* strain XJ-2, XJ-6, XJ-21, and XJ-35 identified in this study. The GenBank accession numbers of 42 strains of *E. canis* were as follows: DJ (North Carolina), DQ146153.1; Florida, DQ146152.1; Cameroon 71, DQ146155.1; Sao Paulo, DQ146154.1; Louisiana, DQ146151.1; Demon, DQ085429.1; Oklahoma, DQ085428.1; Jake, DQ085427.1; Londrina, JX312080; 70C, KF233414.1; 56C, KF233413.1; 1055, KT363877.1; 533, KT363876.1; 36, KT363875.1; CM196, MF771085.1; CM180, MF771084.1; 222, KC479021.1; 171, KC479020.1; 105, KC479019.1; Muzaffargarh3, MH608290.1; Kasur2, MH608289.1; Muzaffargarh2, MH549199.1; Muzaffargarh1, MH549198.1; Rawalpindi2, MH549197.1; Rawalpindi1, MH549196.1; Kasur1, MH549195.1; 46, KT357370.1; 37y97, KT357369.1; Ranana, EU118961; 611, EF636663; Nether, KC935387.1; NGR, JN982341.1; TWN1, EF551366.1; TWN3, EF651794.1; TWN17, HQ009756.1; TWN4, EU139491.1; TWN2, EF560599.1; DB63, MG905720.1; B2-15, MG905719.1; B1-17, MG905718.1; B1-7, MG905717.1; B16, MG905716.1; B12, MG905714.1; DB51, MG905713.1; mkk2, MG905711.1; XJ-2, MN366176; XJ-6, MN366177; XJ-21, MN366178; XJ-35, MN366179.

Phylogenetic analysis based on the amino acid sequence of *gp36* protein showed that *E. canis* strains from different geographical regions could be divided into two gene groups: Genogroup I and Genogroup II (Figure-3), among which the different genogroups could be further classified into different genetic subgroups, showing obvious genetic diversity. The strain XJ-6, together with the Thailand strains (533, 36, and 1055) and the US strain Jake, belonged to subgroup 1.1 of Genogroup I. In contrast, XJ-2, XJ-21, and XJ-35 strains were closely related to the Taiwan strain (TWN1 and TWN4) and the Thailand strain (CM180 and CM196) and belonged to subgroup 2.2 of Genogroup II. The results confirmed that both Genogroup I and Genogroup II epidemic strains of *E. canis* substantively had been circulating in Xinjiang, China.

## Discussion

According to the difference of *16S rRNA* gene, *Ehrlichia* can be divided into five recognized species: *Ehrlichia muris*, *Ehrlichia chaffeensis*, *Ehrlichia ewingii*, *Ehrlichia ruminantium*, and *E. canis* [1]. Among them, *E. ruminantium* mainly infects ruminants (bovine, sheep, goat, etc.), while *E. chaffeensis*, *E. canis*, and *E. ewingii* can infect humans [8]. Accordingly, as a newly discovered pet source zoonotic pathogen, *E. canis* has posed a great threat to public health [9].

In recent years, *E. canis* and its mixed infection with other pathogens have been reported in dogs in many countries and regions around the world [16-19]. Cicuttin *et al*. [11] performed molecular detection of *E. canis* on the canine blood samples from Buenos Aires, Argentina, and the PCR-positive rate was 6.7% (15/223). da Costa *et al*. [15] performed molecular detection of *E. canis* on Brazilian dogs, and the PCR-positive rate ranged from 14.6% to 42.3%. Daramola *et al*. [12] detected blood samples from dogs in South West Nigeria used by nested PCR, and the positive rate was 22.9% (47/2015). Malik *et al*. [13] performed *E. canis* detection on 151 canine blood samples from three regions of Pakistan using used PCR, with a positive rate of 28% (42/151). In this study, it showed that *E. canis* infection had occurred in pet dogs and their skin ticks in Xinjiang. Given that, intimate contact with infected pet dogs could enhance the risks of *E. canis* infection, timely detection, and monitoring of *E. canis* infection in pet dogs and ticks are necessary for public health.

Based on the genome-wide sequence [1,20], many proteins (such as *gp200*, *gp140*, *gp36*, and *gp19*) containing an acidic tandem repeat and an anchor protein repeat interacting with host cell were identified and characterized in *E. canis* [21,22]. *gp36*, as an important antigen protein inducing immune response, belongs to an acidic serine-rich protein with a major antigen epitope in the tandem repeat region. Doyle *et al*. found the variability in the number and sequence of tandem repeat units TRR in *gp36* protein by comparing the *gp36* proteins of the US, Brazilian, and Cameroonian strain of *E. canis* [21]. However, whether selective immune pressure is responsible for the substantial variability in this immunoreactive protein should be further investigated.

According to the genetic variations, *gp36* protein can be used as molecular marker of *E. canis*. Aguiar *et al*. classified *E. canis* into different genotypes: US genotype, Brazil genotype, and the intermediate type [23]. It is worth noting that Bouza-Mora *et al*. identified a new genotype of *E. canis* by genetic analysis of *gp36* protein in the blood samples from Costa Rica blood donors [9]. Maekawa *et al*. [7] confirmed significant differences between *E. canis* strains in the Philippine region and the US and Brazil strains. Furthermore, Nambooppha *et al*. [14] found two gene clusters in the local strain of *E. canis* in the local dog population in Thailand, the USA strain, and the Taiwan strain. In this study, we found that *E. canis* strains from Xinjiang can be divided into two gene groups, which belong to different gene subgroups, respectively, showing substantively genetic diversity.

## Conclusion

This study for the 1^st^ time suggested that Genogroup I and Genogroup II strains of *E. canis* had been circulating in Xinjiang, China, and confirmed that the substantial variability in immunoreactive protein *gp36* had occurred, which provided valuable epidemiological data for the traceability and molecular characteristics of *E. canis* strains from pet dogs.

## Authors’ Contributions

QM, MQ, and QJ planned and designed the whole study. WL, LY, and RY carried out the whole work. CK, ZJ, ZZ, YW, and PY collected sample. QM wrote the manuscript. CX and LC helped during manuscript writing and revision. All authors read and approved the final manuscript.
